# Functional assessment of local versus systemic adipose-derived stromal cell therapy in rodent peripheral nerve regeneration

**DOI:** 10.3389/fneur.2025.1631300

**Published:** 2026-01-12

**Authors:** Stefan Targosinski, Nadine Wieser, Holger J. Klein, Pranitha Kamat, André A. Barth, Elisabeth J. Rushing, Bong-Sung Kim, Jan A. Plock, Riccardo Schweizer

**Affiliations:** 1 Department of Plastic Surgery and Hand Surgery, University Hospital Zurich, Zurich, Switzerland; 2 Faculty of Medicine, University of Zurich, Zurich, Switzerland; 3 Department of Plastic Surgery and Hand Surgery, Cantonal Hospital Aarau, Zurich, Switzerland; 4 Institute of Neuropathology, University Hospital Zurich, Zurich, Switzerland

**Keywords:** adipose derived stromal cells, adipose derived mesenchymal stem cell, peripheral nerve regeneration, sciatic nerve activity, systemic vs. local, cell therapy, route of administration, swim test

## Abstract

**Introduction:**

Adipose-derived stromal cell (ASC)–based therapies may play an important role in peripheral nerve regeneration, though the optimal route of application remains unclear. Systemically injected ASCs have been recognized to home to sites of inflammation and injury but also facilitate a widespread regenerative effect throughout the body. The advantage of local ASC administration is the direct and concentrated application at the site of need. Here, we investigated the effect of local versus systemic ASCs on functional recovery in a rodent sciatic nerve injury model.

**Methods:**

Twenty Lewis rats underwent sciatic transection and repair with a 10 mm nerve autograft and were subsequently assigned into three groups based on the ASC-treatment: systemic (SYS; *n* = 8), local (LOC; *n* = 8) and no ASCs (CTRL; *n* = 4). ASCs (1 × 10^6) were administered i.v. (SYS) or locally at the nerve repair site (LOC). Functional outcome was assessed by a swim test and static sciatic index (SSI) preoperatively and weekly until endpoint (week 14). Gastrocnemius muscle weight ratio and nerve-specific histomorphometry were examined at the endpoint.

**Results:**

The swim test analyses noticed the nerve lesion and assessed functional recovery. Horizontal hindlimb excursion improved slightly and showed earlier recovery in the SYS group compared to LOC at week 6 (*p* < 0.05). The maximum ankle angle increased steadily until the endpoint in the ASC groups, but did not show relevant improvement in controls (CTRL 94.8 ± 10.4° vs. SYS 118.2 ± 19.1°; *p* < 0.001 and LOC 109.8 ± 7.1°; *p* < 0.05). The swim toe spread index (SwTS) and SSI demonstrated recovery from week 6, with superior sensitivity of the SwTS in differentiating between the groups and improved function after systemic administration (CTRL vs. SYS *p* < 0.05). ASCs were able to reduce gastrocnemius muscle atrophy (LOC 0.66 ± 0.04 vs. SYS 0.56 ± 0.18; *p* = 0.18 and vs. CTRL 0.44 ± 0.2; *p* < 0.05). Histomorphometry confirmed axonal remyelination.

**Conclusion:**

Current ASC treatments modestly enhance functional recovery and reduce muscular atrophy after peripheral nerve injury. A slight trend toward better therapeutic effect was observed with systemic administration, although the differences remained small compared to local application. Ideal dosage, optimal timepoint as well as combination of local and systemic ASC-therapies need further assessment.

## Introduction

Peripheral nerve injuries (PNI) cause long-lasting functional deficits that severely impact the quality of life and often lead to long-term inability to work, resulting in a significant burden on both individuals and healthcare systems (1). Such deficits affect the patient’s daily routine and require extensive medical care, rehabilitation and support. Epidemiologic studies in European populations report the prevalence of peripheral nerve injuries range from 11.2 to 13.9 cases per 100.000 individuals annually (2, 3). It is concerning that the rate of complete functional recovery following treatment still remains low (1, 4, 5). The results after direct coaptation are often unsatisfying and are even worse when nerve grafts or conduits are required (1, 6). Nerve regeneration is a complex process, yet we know that acceleration of axonal growth results in improved functional outcome (7, 8). Different strategies are being investigated to promote nerve regeneration, including cell-based therapies with mesenchymal stromal cells (MSCs) or mesenchymal stem cells derived from bone marrow or adipose tissue (9–15).

Adipose-derived stromal cells (ASCs) have shown several benefits, notable the ease of harvesting large numbers via liposuction with minimal donor-site morbidity (16, 17). ASCs exhibit promising therapeutic effects in peripheral nerve regeneration owing to their capacity to differentiate into various cell types, including nerve cells and their ability to secrete growth factors that stimulate nerve growth and regeneration. The multifaceted properties of ASCs encompassing differentiation into Schwann cells, secretion of growth factors like neurotrophin nerve growth factor (NGF), brain-derived neurotrophic factor (BDNF), and glial cell-derived neurotrophic factor (GDNF), promotion of angiogenesis, and anti-inflammatory effects highlight their role in nerve regeneration (9–12, 18).

Two methods of ASC administration for peripheral nerve regeneration, namely local and systemic administration, have been suggested in the past (10, 19–24). Local administration entails direct injection into the injury site, offering a concentrated cell source and potentially improved efficacy. Challenges remain regarding invasiveness of the procedure and delivery precision (10, 19, 20). Systemic administration via intravenous injection provides less invasive delivery. This method may lead to lower cell concentrations at the injury site and potential clearance from the body before reaching the site of injury. However, it may trigger paracrine effects that indirectly support regeneration (21–24). Both local and systemic administration of ASCs have shown promising results in experimental studies for peripheral nerve regeneration. The present study therefore compares the optimal method of administration in a rodent sciatic nerve injury model with comprehensive functional and descriptive outcome analysis.

## Methods

This study was performed in accordance with the Swiss animal welfare ordinance and was approved by the local animal experimentation committee (Zurich, Switzerland; permission ZH 181/2018).

### ASC isolation, cell culture and characterization

Inguinal fat pads from Lewis rats were processed using enzymatic and culturing methods to isolate and characterize cell populations according to previously applied protocols (24). In short, ASCs were isolated from adipose tissue excised from the inguinal fat pads of anaesthetized Lewis rats under sterile conditions. The tissue was minced and digested with collagenase type II (Worthington Biochemical Corp., USA) and bovine serum albumin (Millipore, USA) in Hanks’ balanced salt solution for 60 min at 37 °C under gentle agitation. After washing, erythrocyte lysis, and filtration through sterile gauze, the cell pellet was cultured in DMEM/F12 medium (Gibco, USA) supplemented with 10% fetal bovine serum, 0.1 μM dexamethasone, and 1% penicillin–streptomycin. Non-adherent cells were removed after 6 h, and adherent ASCs were expanded to passage 2 before use. Flow cytometry confirmed the mesenchymal phenotype (CD45^−^, CD29^+^, CD73^+^, CD90^+^). For the present study, the ASCs were harvested from donor syngeneic animals in previous experiments, which were cryoconserved at −80 °C and banked until use. For application, the cells were thawed, 1 × 10^6^ ASCs were prepared in sterile PBS and administered either systemically (intravenously; SYS) or locally at the nerve repair site (LOC) during surgery.

### Groups and experimental protocol

**Figure 1:**
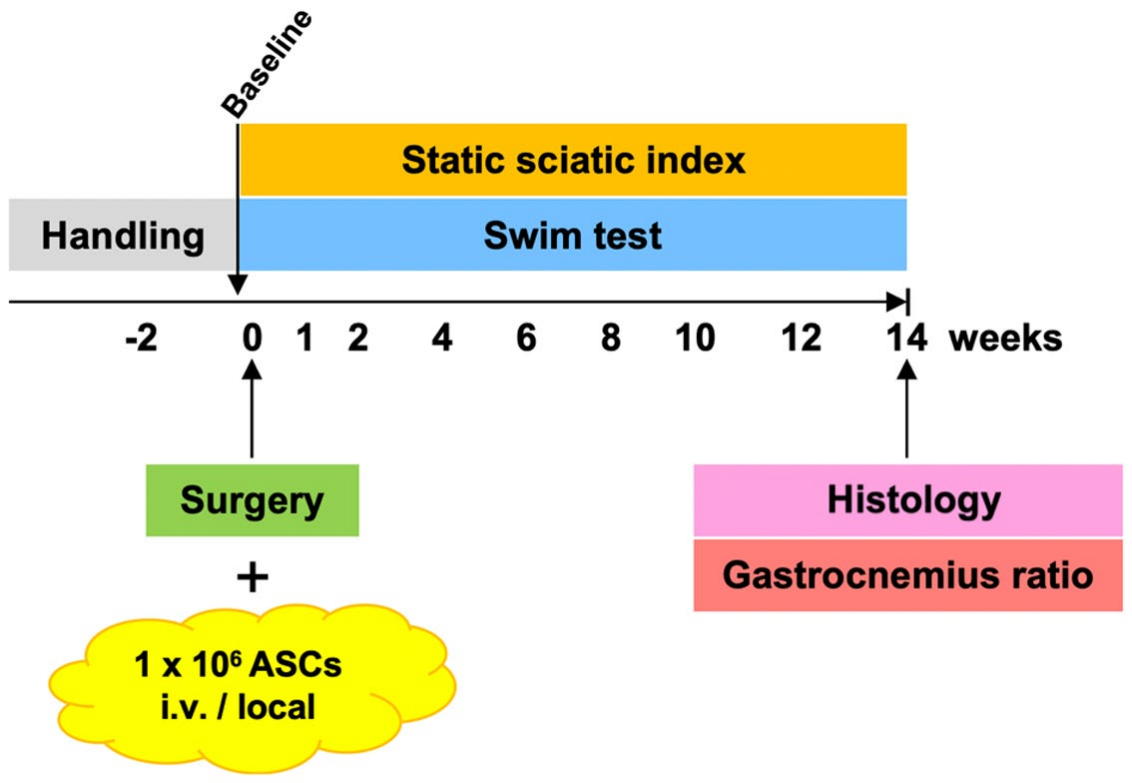
Study protocol: the cells were administered during surgery, and functional assessments were performed for 14 weeks on a weekly to biweekly basis after acclimatization, handling, and capturing a preop baseline.

Twenty 6- to 8-weeks-old male Lewis rats (200–250 g, Janvier/Charles River, Germany) were included in the study and were randomly assigned to three groups based on the ASC treatment: systemic (SYS; *n* = 8), local (LOC; *n* = 8) or no ASCs (CTRL; *n* = 4). ASCs (1 × 10^6^) were administered intravenously (SYS) or locally at the site of the operated nerve (LOC) during surgery. The animals were acclimatized in the animal facility for 2 weeks prior to handling and familiarization to the functional assessment apparatus. Before surgery baseline (BL) assessment was conducted. Postoperative functional outcome was analyzed at weeks 1, 2, 4, 6, 8, 10, 12, 14 (Figure 1). At postoperative week 14, following functional assessment, rodents were euthanized and tissue samples were harvested for further histological analysis. To ensure unbiased assessment, a systematic coding was performed ensuring blinded analysis of images and measurements across the experimental groups.

### Surgical procedure

Surgeries were performed under isoflurane-inhalation-anesthesia, which was induced by placing the animal into an induction chamber with 4–5% isoflurane and was maintained with 2–3% isoflurane (100% oxygen at a flow rate of 0.3 L/min) through a snout mask. The hindlimb was shaved and then disinfected three times. Local analgesic was administered using 1–2 mL Lidocaine (1%). A skin incision was made and through careful blunt dissection, the sciatic nerve was exposed using a dorsal approach through the thigh musculature. A 10-mm long nerve graft was harvested from the sciatic nerve, followed by a microsurgical epineural repair with 9–0 nylon sutures (Ethicon LLC, USA), sutured back in a reversed fashion with epineural sutures on both ends. Then, a single dose of 1 × 10^6^ ASCs was administered intravenously through the dorsal penile vein (SYS) or added locally to the site of the operated nerve (LOC). No cells were administered in the control group. Skin closure was performed with running with 4–0 resorbable braided sutures (Vicryl Rapid, Ethicon LLC, USA). Wounds were covered with Opsite® Spray Dressing (Smith&Nephew, UK). All microsurgical procedures were performed by experienced microsurgeons under magnification with an operating microscope (Zeiss, Germany). After the surgery the animals were carefully observed for recovery. Postoperative pain control was ensured by administration of subcutaneous injection of buprenorphine (Temgesic®; 0.1 mg/kg) with 1 mL Ringer’s lactate for hydration. In addition, analgesics were provided in drinking water for at least 72 h (paracetamol; Dafalgan Sirup®; 200 mg/mL).

### Functional assessment

**Figure 2:**
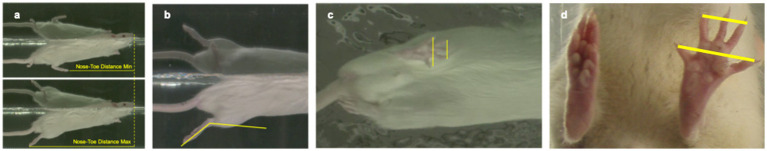
Functional assessment: swim test parameters: **(a)** Horizontal hindlimb excursion, **(b)** maximal ankle angulation, **(c)** swim toe spread index, and **(d)** static Sciatic Index (SSI). For the SSI: the rats were positioned in a transparent Plexiglas chamber while pictures were taken from underneath using a camera at a fixed distance from the box’s base when both hind paws were placed on the ground. The widths of toe spread (TS; first to fifth toe) and intermediary toe spread (ITS; second to fourth toe) on the operated (O) and non-operated (N) sides were measured. The SSI formula was used as defined previously (26): SSI = 108.44 (OTS – NTS) / NTS + 31.85 (OITS – NITS) / NITS – 5.49.

Dynamic functional parameters were assessed using a swim test as described previously by Targosinski et al. (25). Recordings of three selected swims were analyzed for horizontal hindlimb excursion (Figure 2A), maximum angulation in the ankle joint (Figure 2B) and the swim toe spread index (SwTS) (Figure 2c). Static functional parameters were assessed utilizing the Static Sciatic Index (SSI) as conducted by Bervar (Figure 2d) (26). The findings were displayed using percentages (%), where 0% indicated no functional impairment, and a value of −100% denoted a total loss of function. The open-source software Fiji (ImageJ, NIH, USA); (44) was used for the measurements of the dynamic and static parameters.

### Euthanasia

At the endpoint, the animals were euthanized in a carbon dioxide-filled chamber (flow rate 3 L/min) for at least 15 min, until cardiac and respiratory arrest were confirmed. Subsequently, nerve and muscle samples were harvested for further analyses.

### Gastrocnemius muscle ratio

The wet weight of the gastrocnemius muscles was determined after tissue sampling at the endpoint. The gastrocnemius muscle ratio was calculated by dividing the wet weight of the gastrocnemius muscle of the operated side divided by the contralateral side.

### Histological analysis

At the endpoint, nerve segments were excised immediately distal to the autograft neurorrhaphy, which was identified by a nylon suture. Analogous sections were obtained from the non-operated side at an equivalent level. Preparation of semi-thin cross-sections with toluidine blue staining of the nerve tissue was performed. The cross-sections were imaged at a 10x magnification for overview pictures and a 63x magnification with immersion oil for detailed analysis using a Leica DM6000-B microscope (Leica Microsystems GmbH, Germany). The microscope imaging software LAS X software (Leica Microsystems GmbH, Germany; Version 3.7.4.23463) was used for digitalization and capturing of the images. Five representative regions (100 × 100 μm) of each nerve cross-section were analyzed with Fiji (ImageJ, NIH, USA). Fiber density was expressed as the number of fibers per 10^4^ μm^2^ cross-sectional area. Nerve fiber (FD) and axon diameter (AD) were measured. Calculation of the myelin thickness (MT) involved the application of the formula (FD-AD)/2. The g-ratio was determined by dividing AD by FD (27).

### Statistical analysis

Data were analyzed using Prism 8.0 (GraphPad Software, USA). The means, along with their respective standard deviations (SD), were computed for each functional and histological parameter within the experimental groups (means ± SD). Statistical significance of the recovery in horizontal excursion and maximum angle, a 2-way ANOVA with Dunnett’s multiple comparison test was conducted, analyzing both the intergroup and intragroup variations across different time points. Comparison of nerve parameters from cross-sections distal to the autograft with those of the healthy leg, was achieved using 2-way ANOVA to ascertain statistical significance. For the assessment of differences among groups pertaining to each histological parameter and the gastrocnemius muscle ratio, one-way ANOVA was employed. Significance thresholds were set as * *p* < 0.05, ** *p* < 0.01, *** *p* < 0.001, and **** *p* < 0.0001.

## Results

All 20 rodents reached the designated endpoint at postoperative week 14. Impaired hindlimb function was observed in all operated subjects immediately after the animals awakened from anesthesia. The study was performed without wound healing complications, infections or unexpected weight loss. Furthermore, there was no clinical evidence of neuroma formation throughout the experiment.

### Functional recovery

A notable functional deficit in horizontal excursion, maximum ankle joint angulation, swim toe spread index (SwTS) and the SSI was found in all groups at week 1 following the intervention.

#### Horizontal hindlimb excursion in mm

**Figure 3:**
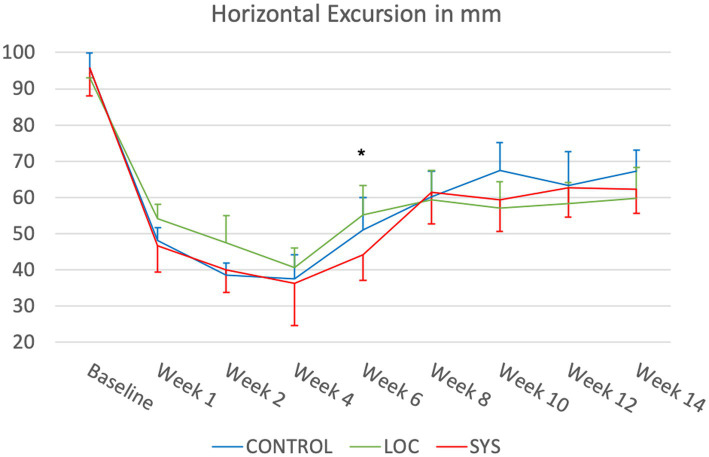
Horizontal hindlimb excursion. Significance was defined as *p* < 0.05; LOC vs. SYS.

At baseline (BL), the CTRL group exhibited a mean horizontal excursion of 95.47 ± 4.36 mm, while the LOC group had a mean of 92.96 ± 3.80 mm, and the SYS group had a mean of 95.77 ± 7.84 mm (Figure 3). Horizontal excursion showed a drop in the first postoperative week in all groups (CTRL 48.11 ± 3.60 mm; LOC 54.22 ± 7.45 mm; 46.63 ± 7.34 mm). From weeks 2 to 4 the decline continued with the CTRL, LOC, and SYS groups demonstrating the lowest mean values of 37.39 ± 6.70 mm, 40.66 ± 7.96 mm and 36.29 ± 11.61 mm. At week 6 LOC showed a significant improvement against the systemic administration group (*p* < 0.05). The LOC group showed a minimal but earlier improvement until week 8, which did not persist at later time points. All groups exhibited modest increases by week 8 with similar regain of function (CTRL 60.21 ± 7.08 mm; LOC 59.39 ± 7.22 mm; SYS 61.42 ± 7.06 mm). Slight variations persisted throughout the groups but did not result in substantial deviations between weeks 8 to 14. At the endpoint, the CTRL group exhibited a mean value of 67.28 ± 5.69 mm, the LOC group had 59.84 ± 7.33 mm and the SYS group maintained at mean value of 62.17 ± 6.53 mm. A full return of function was not achieved for horizontal hindlimb excursion during this time frame.

#### Maximum ankle joint angulation

**Figure 4:**
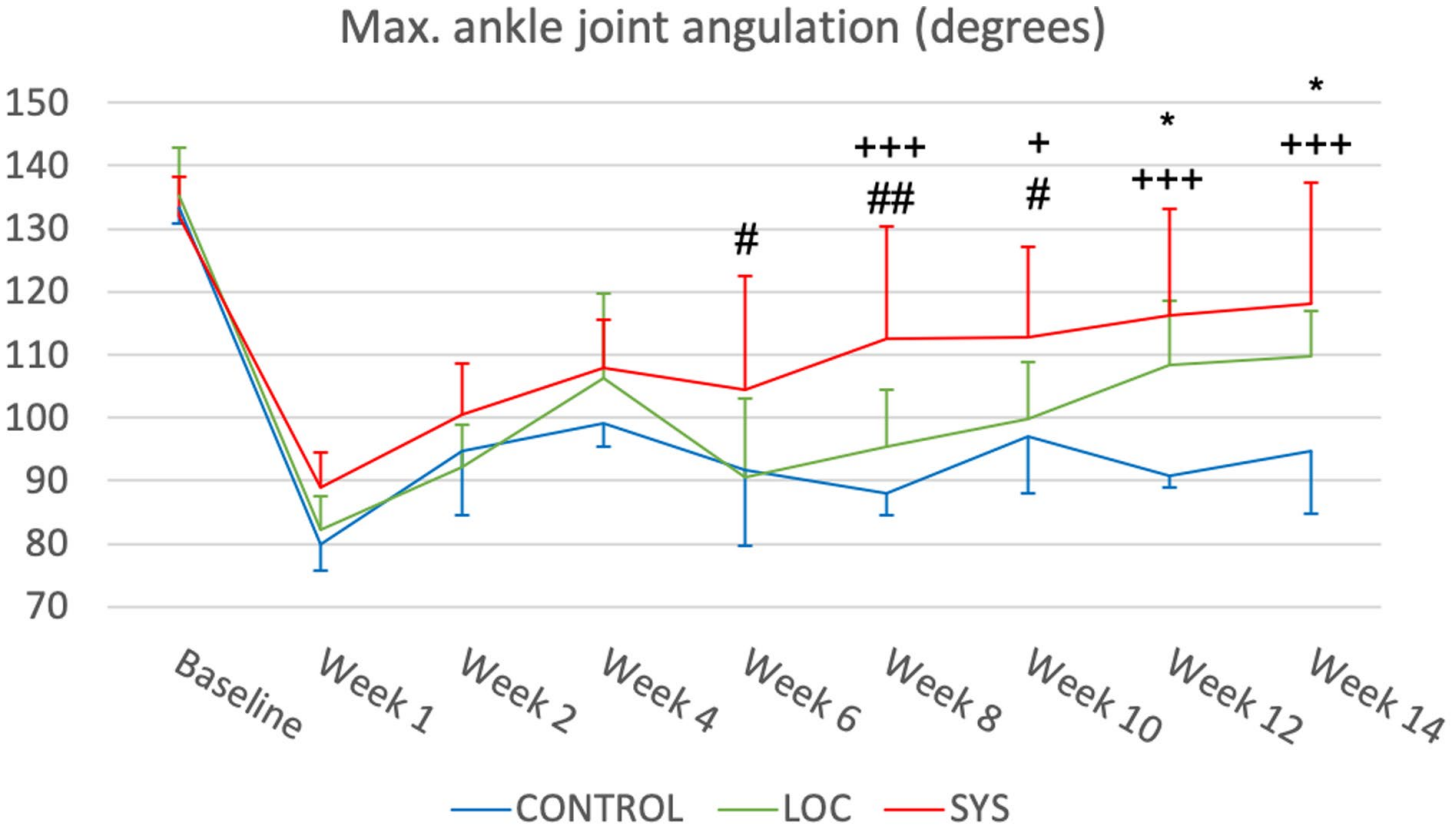
Maximum ankle joint angulation. Significance was defined as *p < 0.05, ** *p* < 0.01, ***p < 0.001; * CTRL vs. LOC; +CTRL vs. SYS; #LOC vs. SYS.

At BL, the mean maximum ankle joint angulation for CTRL was 133.34° ± 2.51°, whereas LOC was 135.27° ± 7.48° and SYS 132.03° ± 6.14° (Figure 4). Following surgery at week 1, ankle motion decreased to a maximum angle of 79.89° ± 4.10° in CTRL, 82.21° ± 5.39° in LOC and 88.94° ± 5.50° in SYS group. All groups exhibited gradual increases in ankle joint angulation to week 4. Throughout week 6 to week 14, gradual increases were observed across the treatment groups. At week 8, CTRL showed 97.04° ± 3.61°, LOC 99.81° ± 9.01° and SYS a marked rise to 112.60° ± 18.11°. SYS consistently demonstrated significantly higher ankle joint angulation compared to the other groups. Toward the endpoint at week 14, CTRL reached 94.80° ± 10.04°, LOC showed 109.77° ± 7.06° and SYS displayed the highest value at 118.18° ± 19.07°. Notably, the differences between CTRL and both LOC and SYS groups were significantly pronounced (CTRL vs. LOC: *p* < 0.05; CTRL vs. SYS: *p* < 0.001).

#### Swim toe spread index (SwTS)

**Figure 5:**
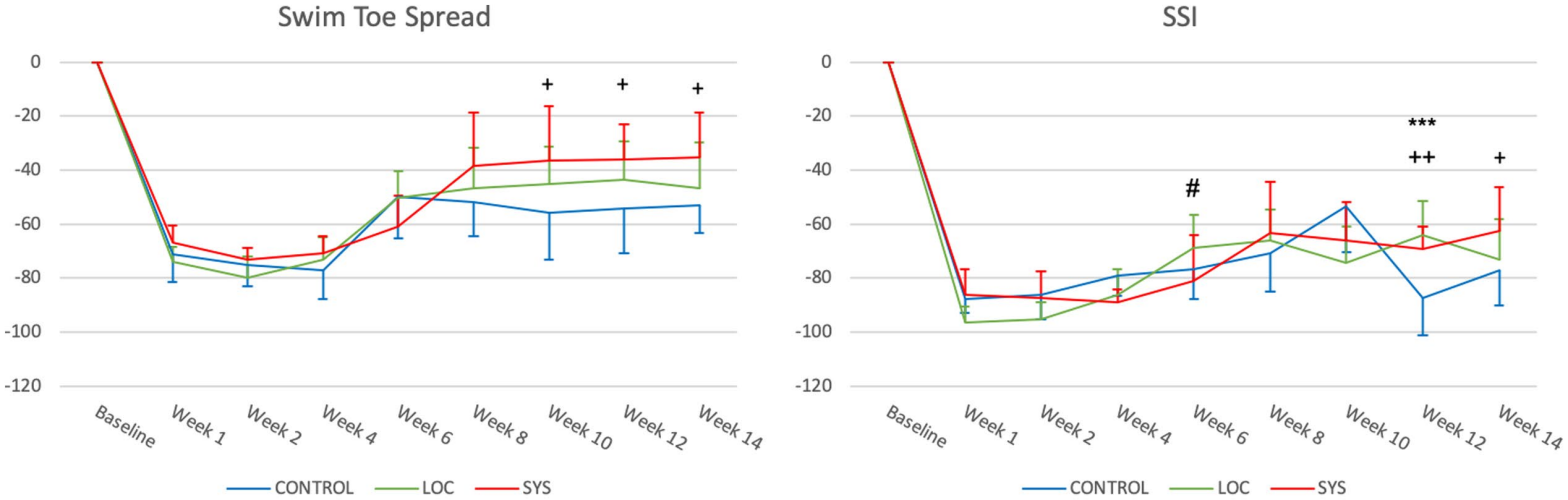
Swim toe spread index (SwTS) and static sciatic index (SSI). Significance was defined as *p < 0.05, **p < 0.01, ***p < 0.001; *CTRL vs. LOC; + CTRL vs. SYS; # LOC vs. SYS.

Normal hindlimb function is represented as “0” for the preop BL. At week 1, the CTRL group exhibited a decrease to −71.27 ± 10.27, while the LOC and SYS groups showed similar values of −73.76 ± 5.15 and −66.80 ± 6.43 (Figure 5). In the subsequent weeks, marginal changes were observed among the groups (week 4 CTRL −77.20 ± 10.64, LOC −73.35 ± 8.64 and SYS −70.89 ± 6.44). At the 6th postop week, all groups showed a notable upward deviation with a mean value of −49.99 ± 15.19 in the CTRL, −50.23 ± 9.99 in the LOC and −60.93 ± 11.55 SYS group. The values for the CTRL group remained low and relatively stable with only minimal improvement until week 14 to a mean of −53.20 ± 9.93. LOC showed a similar trend until the endpoint of the functional tests (−46.65 ± 17.13). The SYS group continued to increase (−35.29 ± 16.75 at week 14) and demonstrated significance compared to the no ASC-group from week 10 to 14 (*p* < 0.05).

#### Static sciatic index (SSI)

All groups displayed a remarkable drop in postoperatively at week 1 (CTRL -87.78 ± 5.20, LOC -96.62 ± 5.91, SYS -86.20 ± 9.44) (Figure 5). In the CTRL group, the SSI gradually increased to −53.61 ± 16.63 at Week 10, followed by a notable drop at week 12 (−87.31 ± 16.63) and a mean value reaching to −77.04 ± 12.92 at the endpoint. LOC showed earlier improvement compared to SYS at week 6 (*p* < 0.05). However, this climb in the LOC group stabilized toward the later weeks: −68.71 ± 12.02 (week 6), −66.22 ± 11.49 (week 8), −74.19 ± 13.21 (week 10), −63.91 ± 12.34 (week 12), and −72.99 ± 14.85 (week 14). Following week 6 (−80.90 ± 16.73) SYS group demonstrated a further increase and slightly superior function across the rest of the assessment period to −63.09 ± 18.75 at week 8, −65.94 ± 14.23 (week 10), reaching significance at week 12 (−69.22 ± 8.40; *p* < 0.01) and at week 14 (−62.31 ± 15.85; *p* < 0.05).

#### Gastrocnemius muscle weight ratio

**Figure 6:**
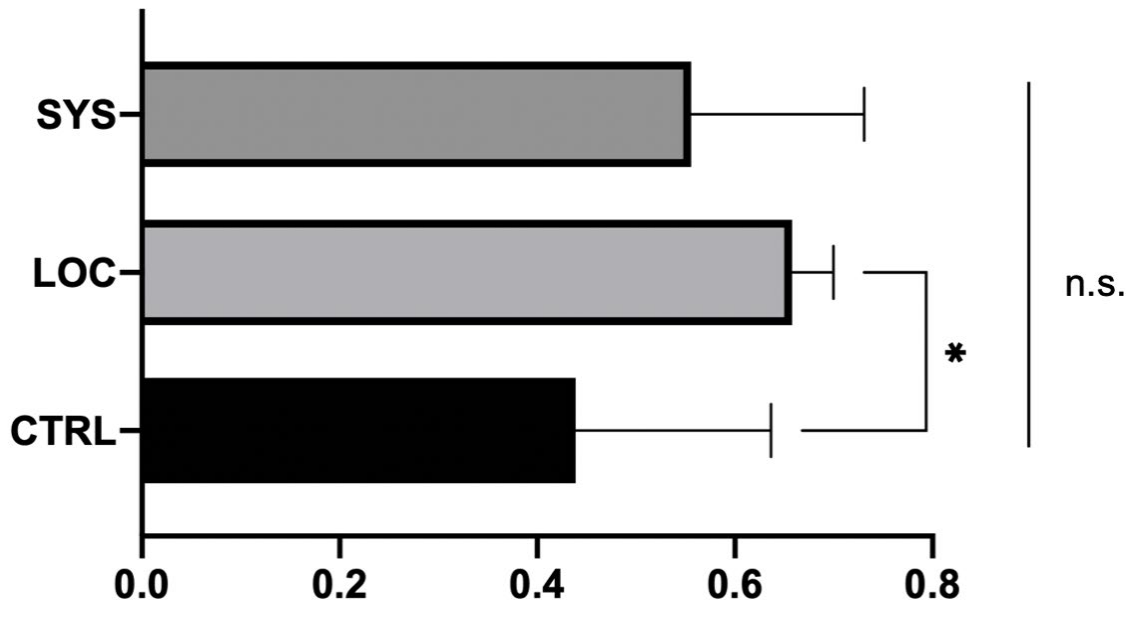
Gastrocnemius muscle weight ratio revealed a decreased muscle atrophy in the local ASC group, whereas a significant muscle atrophy was observed in the CTRL group and the wet weight was slightly decreased in the SYS group. *p* < 0.05; CTRL vs. LOC.

ASC therapy showed a reduced postoperative muscle atrophy in LOC (0.66 ± 0.04; p < 0.05) and SYS (0.56 ± 0.18; *p* = 0.18) compared to CTRL (0.44 ± 0.20) (Figure 6).

#### Histology

**Figure 7:**
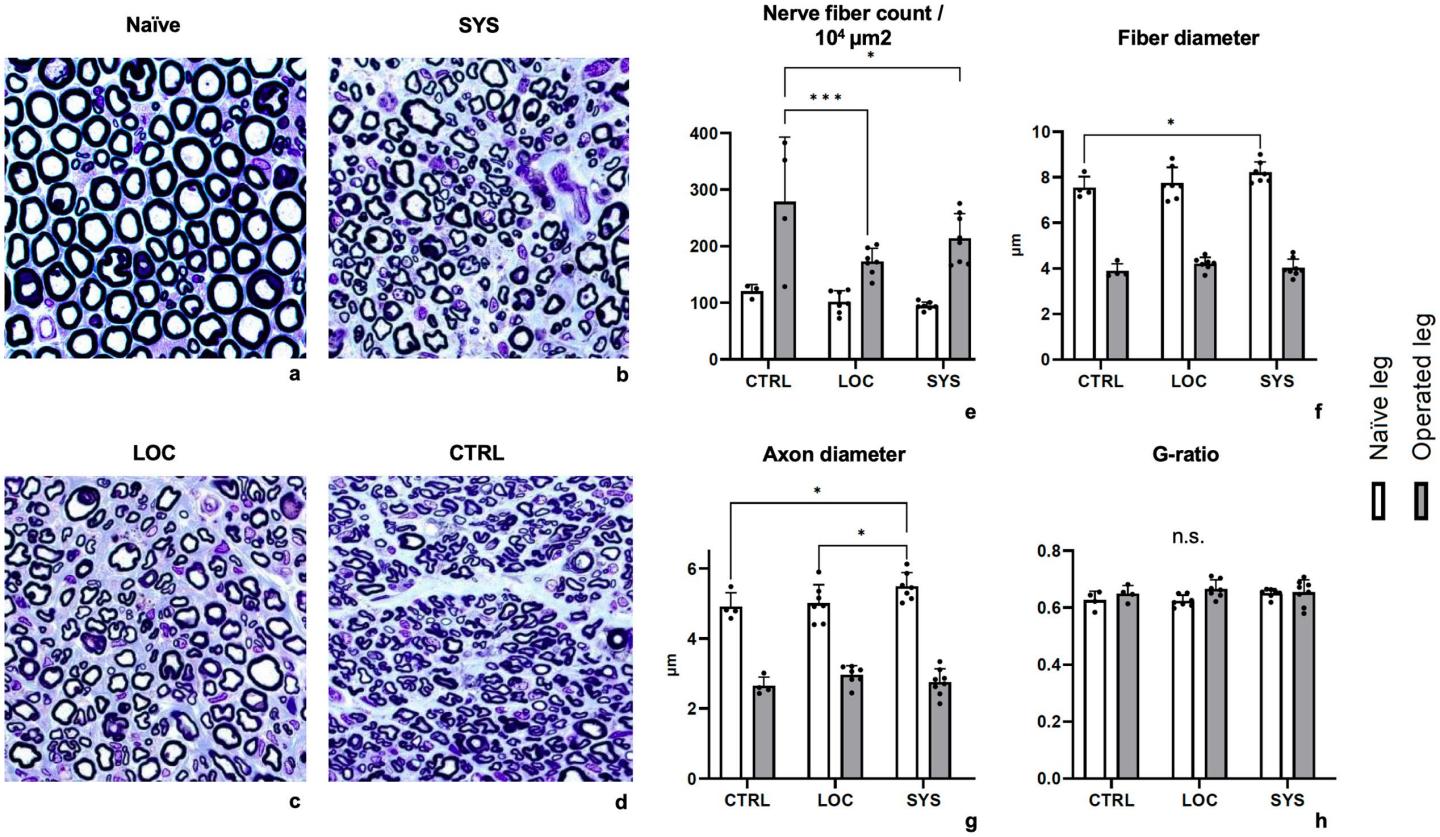
Nerve histomorphometry: Examples of 100 × 100 μm nerve cross-sections of a naïve sciatic nerve and distal to the autograft following nerve specific staining with Toluidin blue (**(a)** Naïve, **(b)** SYS, **(c)** LOC, **(d)** CTRL). Nerve specific histomorphometry confirmed axonal remyelination (**(h)** g-ratio). All g-ratio measures remained within the normal physiological range for regenerating fibers at the endpoint. **(e)** Nerve fiber count/10^4μ^m^2^, **(f)** Fiber diameter, **(g)** Axon diameter, **(h)** g-ratio. * *p* < 0.05, *** *p* < 0.001.

Nerve fiber density was highest in the CTRL group (278.05 ± 108.34 per 10^4 μm^2), followed by SYS (217.30 ± 47.94 per 10^4 μm^2) and LOC (172.74 ± 28.36 per 10^4 μm^2). Fiber density in both treatment groups was significantly reduced compared with CTRL (CTRL vs. SYS *p* < 0.05; CTRL vs. LOC *p* < 0.001) (Figure 7e). Fiber diameter (FD) (Figure 7f) showed comparable mean values across all groups (CTRL 3.80 ± 1.75 μm; LOC 4.18 ± 2.13 μm; SYS 3.96 ± 1.87 μm), with LOC presenting the widest distribution. Axon diameter (AD) demonstrated a similar pattern (CTRL 2.56 ± 1.46 μm; LOC 2.93 ± 1.84 μm; SYS 2.68 ± 1.57 μm) (Figure 7g). All g-ratios (CTRL 0.64 ± 0.11, SYS 0.65 ± 0.11 and LOC 0.66 ± 0.12) remained within the physiological range for regenerating peripheral nerves and indicated ongoing axonal maturation and remyelination (Figure 7h).

## Discussion

This study investigated the potential therapeutic effect of ASCs in peripheral nerve regeneration with special focus on the route of local and systemic administration in a rodent sciatic nerve injury model. Animals receiving systemic ASC administration (SYS) were treated intravenously, whereas those in the local group (LOC) received ASCs directly at the nerve repair site. The CTRL group underwent the same nerve repair without ASC administration. These two delivery routes represent distinct therapeutic approaches and understanding this distinction is essential when discussing the functional and structural outcomes observed. We employed a refined swim test setup, previously validated by our group, to provide reliable functional outcome measures using multiple parameters (25). This study provides a detailed analysis of functional recovery in rodents post-surgery, using several parameters like horizontal hindlimb excursion, ankle joint angulation, swim toe spread index and the static sciatic index. The data clearly demonstrate an initial post-surgical drop in function in all groups, followed by varying degrees of recovery over time. Notably, all groups showed incomplete recovery and remained below normal baseline levels throughout the study period. Within this overall limited recovery, the SYS group demonstrated slightly better performance in several parameters, including maximum ankle joint angulation, SwTS and SSI, although these differences remained modest and did not indicate full functional restoration. The LOC group showed early improvements in horizontal hindlimb excursion, suggesting localized treatment might accelerate initial recovery. However, while there were slight improvements in all groups, none of the groups returned to their baseline levels and displayed similar levels of incomplete recovery in the follow up period. This pattern suggests that while local administration may transiently accelerate early recovery through concentrated trophic effects at the repair site, systemic delivery provides broader and more durable paracrine and immunomodulatory support, resulting in more consistent long-term functional benefits.

LOC performed slightly weaker in ankle joint angulation than the SYS, but showed a significant increase in motion over the control group at later timepoints. Finishing with the lowest angulation, CTRL emphasizes the significant lack of joint mobility restoration.

Despite an initial promising trend of LOC in the SwTS and the SSI, the LOC group’s results almost converged with those of SYS, indicating limited long-term benefits. In the CTRL group, a slightly lower but still the least progress was seen for recovery. The SSI showed fluctuating and inadequate recovery trends in the controls, reflecting deficits in nerve function. These findings support cell-based therapy in peripheral nerve regeneration; however, the advantage is very limited with the dosing and timepoints used in this study. Our results suggest that systemic administration of the treatment might be slightly more effective in enhancing functional recovery post-surgery.

Similar to our functional results, the ASC groups revealed decreased muscle atrophy compared to CTRL. The gastrocnemius muscle weight ratio showed that local ASC administration significantly reduced muscle atrophy compared with controls, whereas systemic treatment produced only a modest, non-significant effect. This suggests that local delivery may exert stronger short-term trophic influences on muscle preservation, likely by providing concentrated support directly at the repair site. However, this benefit did not translate into superior long-term functional outcomes, where systemic therapy was slightly more effective. Thus, while LOC may protect muscle mass early on, SYS appears to promote broader neural recovery processes that ultimately support function more consistently. Although both ASC treatments mitigated postoperative muscle atrophy compared to controls, the difference between local and systemic administration was not statistically significant. This indicates that preserved muscle mass does not necessarily translate into improved functional performance once reinnervation has occurred. The early trophic support provided by local ASCs may have reduced initial denervation-related atrophy, whereas systemic ASCs likely promoted more sustained neural regeneration through paracrine and immunomodulatory pathways, resulting in superior long-term function. Since muscle weight was analyzed only at 14 weeks, when the nerve gap was already regenerated, earlier measurements might have revealed transient advantages of local delivery that were no longer evident at the endpoint.

In histomorphometry, local application showed slightly lower fiber counts than SYS and significantly less than CTRL, but is higher compared to the naïve nerve of the contralateral side. The nerve size parameters remain similar in all groups, which may correlate with advanced maturity of nerve regeneration after 14-weeks (Figures 7e–h). As regenerated nerves have smaller diameters, the fiber count per defined cross-sectional area can appear higher on the operated side than in naïve nerves (Figure 7e). Ronchi et al. explicitly note that regenerating fibers branch and can increase in number distally to the lesion, with fiber counts later returning toward normal as pruning and maturation proceed (28). Similarly, Fox et al. reported mean myelinated fiber counts in the regenerated rat sciatic nerve exceeding those of normal nerves at early time points after end-to-end repair, despite a stable motor neuron pool (29). Transiently elevated distal fiber counts are a well-documented characteristic of early peripheral nerve regeneration, driven by collateral sprouting and multiple axonal branches that increase the number of myelinated profiles before pruning restores more physiological architecture (7). In addition, regenerating axons often branch during the early phase of regeneration to increase the likelihood of target reinnervation, followed by pruning and maturation over time. Therefore, a high distal fiber count reflects active sprouting (7, 28–30). The fiber density in the ASC groups showed a significant lower nerve count which may indicate more advanced maturation, consistent with physiological g-ratio values and functional recovery outcomes. Since the primary objective of this study was functional assessment, histomorphometry was included mainly for structural correlation. Further, histomorphometric analysis confirmed axonal remyelination in all groups. The g-ratio within a physiologic range in all groups should be viewed as an indicator of active remyelination and axonal maturation. This is supported by the absence of morphological abnormalities and the modestly improved functional recovery. The difference, although small, suggests a more advanced stage of regenerative remodeling under systemic ASC therapy, consistent with proved axonal growth, but which was similar to the other groups. These findings align with prior studies demonstrating the potential of ASCs in nerve regeneration, highlighting their ability to transdifferentiate into various cell types including Schwann cell-like cells (SCLCs), to secrete growth factors or exosomes and promote angiogenesis as well as their anti-inflammatory effects (9–12, 16–18, 31–34).

The majority of research conducted in this field has investigated the effectiveness of local ASCs applied directly to the site of the nerve lesion, e.g., integrated in scaffolds such as conduits, gels or conditioned media (20, 24, 35, 36). Saller et al. described the utility of coating autografts with fibrin-encased ASCs and showed a slight but apparently significant improvement in structural and functional outcomes in the presence of the stromal cells (35). Another study presented comparable results with an epineural tubulization together with ASCs injected inside the tube in a rodent sciatic nerve transection model that exhibited enhanced functional outcome along with peripheral axonal regeneration (36). In an *in vitro* and *in vivo* study utilizing a sciatic transection and repair model with an exosome-gel mixture placed inside a silicone tube, ASC-derived exosomes promoted nerve regeneration by stimulating Schwann cell function including secretion of neurotrophic factors (34). Furthermore, Tremp et al. expedited nerve healing by utilizing ASCs for epineural injection, which promoted angiogenesis, transdifferentiating into SCLCs and released neurotrophic growth factors (20). Our study supports these findings with local ASC application resulting in improved functional and morphological outcomes in most parameters compared to the control group. However, local application presents certain challenges, including the risks of additional damage at the injury site, diffuse distribution as well as survival of transplanted cells in a diseased environment (18, 37, 38).

Alternatively, it has been shown that systemically injected MSCs home to sites of inflammation and injury due to the action of local cytokines and chemokines (39). Their ability to migrate to the site of injury qualifies ASCs as potential candidates for systemic administration. Still, only a few studies have investigated the effects of systemic ASC administration on peripheral nerve regeneration. In another sciatic nerve crush injury model, ASCs were applied intravenously through the tail vein 1 week postoperatively. This group reported improvement of axonal outgrowth due to the action of neurotrophic factors and a downregulation of inflammatory markers in the presence of undifferentiated ASCs migrated to the site of the nerve lesion (23). In the context of vascularized composite allotransplantation (VCA) systemically and locally applied ASCs were compared with regard to their immunomodulatory and vasoprotective effects during acute rejection in a rat hindlimb transplantation model (40). They have shown that both forms of application were able to modulate inflammatory activity and reduce vascular damage to promote graft tolerance, but were not sufficient to stop rejection without additional immunosuppression. These results confirm the importance of systemic paracrine effects and demonstrate the therapeutic efficacy of ASCs for localized action. Combined or sequential application strategies as well as optimized dose and time regimes could offer additional therapeutic benefits in nerve regeneration and for graft protection in VCA (40). The effects of local as well as systemic mesenchymal stromal cells (MSC) were analyzed in a rodent sciatic nerve cut and repair and hindlimb transplant model. Rats treated with MSCs through either local or systemic injections showed marked enhancement in the recovery rate of compound muscle action potential amplitudes and axon numbers compared to control groups. In cases of allogeneic hindlimb transplants, rats receiving MSCs via local injections demonstrated a notable increase in axon numbers (41). Systemic MSC administration led to better nerve regeneration after hindlimb transplantation. The systemic approach was more effective in boosting electromotor recovery, whereas local injections primarily increased fiber counts, indicating distinct mechanisms of action. Both local and systemic applications of MSCs significantly accelerated and intensified nerve regeneration following nerve injuries and hindlimb transplants (41). Schweizer et al. have proven the safety and efficacy of systemic administration of ASCs post nerve transection. The effectiveness of systemic ASC administration was investigated, revealing that systemic application modestly improves functional outcomes compared to the control groups receiving no cell treatment (24). Our findings extend those of Schweizer et al., as this study combined a more demanding 1-cm reversed autograft model with extended follow-up period and multiple functional readouts with a more refined setup, allowing us to demonstrate both a transient early advantage of local delivery and the more consistent long-term benefit of systemic administration. Their study was important not only for its findings on systemic administration of ASCs but also for establishing an improved swim test for functional assessment in peripheral nerve regeneration. The swim test evaluates motor function and coordination by observing the rodent’s ability to swim and navigate through water, reflecting the recovery of nerve function (24, 25, 42). Our research group further explored the swim test as a functional analysis method in rodent models, confirming its reliability as an assessment tool for peripheral nerve regeneration. This method complements the commonly used SSI and histological analyses, offering a broader perspective on treatment outcomes.

The study’s methodologies, particularly the use of different functional and histological measures, are robust and provide a comprehensive overview of the regeneration process. However, limitations based on the animal model used and specificities of the surgical and treatment procedures might impact translating the results to human patients. The animals were 6–8 weeks old at the start of the experiment and showed a physiological increase in body weight over 14 weeks. As this growth occurred uniformly across all groups, it did not affect between-group comparisons. Normal post-adolescent development may have a minor influence on morphometric parameters and represents a general limitation of long-term studies in growing animals.

In this study, syngeneic rather than autologous ASCs were used. The use of a syngeneic donor avoids effects due to immunological rejection while preventing an additional surgical intervention in the same animal, which would have represented an additional trauma and could have influenced the outcomes of functional recovery after nerve injury (e.g., homing of cells to site of cell harvesting). Additionally, to harvest the highest number of cells out of rat donors, usually both inguinal fat pads are harvested, which would have compromised the surgical field for the nerve cut and repair model due to proximity. Although this approach does not fully replicate a true autologous clinical scenario, it “simulates” an autologous approach and allows for a controlled evaluation of the therapeutic effect of ASCs without bias related to donor site injury and/or immunological effects.

Following intravenous administration, an initial accumulation in the lungs was described that suggests a potential filtering or trapping mechanism (22). This raises questions about the longevity and systemic distribution of these cells. In systemic applications, the administration of ASCs may require increased and more frequent dosages. The goal of this approach is to overcome the first-pass effect of filtering organs, which can reduce the cell concentration reaching target tissues. Optimizing dosage is crucial for enhancing the therapeutic effectiveness of ASCs (43). Additionally, there are potential safety concerns related to the use of ASCs, such as the risk of tumor formation or immune rejection, which must be addressed before application in clinical practice. Despite these challenges, promising results of ASC therapy in preclinical studies indicate the potential for use in the future treatment of peripheral nerve injuries.

## Conclusion

ASCs and the route of administration impact the efficacy of the treatment by functional and histological outcomes in a peripheral nerve injury model. A subtle trend favoring systemic administration was seen in several outcome measures, suggesting that the systemic distribution of ASCs might offer a more comprehensive therapeutic approach, possibly addressing different mechanisms in the nerve regeneration process and thus promoting better overall recovery following nerve injury. However, these trends were modest and should be interpreted cautiously. In addition, the study highlights the advantages of the swim test for the post-surgical functional assessment of nerve recovery in rodents. Future research should focus on optimizing ASC treatment protocols addressing such aspects as dosage, timing and/or the combination of systemic and local application in order to validate their efficacy and safety in human patients.

## Data Availability

The original contributions presented in the study are included in the article/Supplementary material, further inquiries can be directed to the corresponding author.
