# Supplementary material for: Functional assessment of local versus systemic adipose-derived stromal cell therapy in rodent peripheral nerve regeneration
Source: Front Neurol. 2026 Jan 12;16:1631300. doi: 10.3389/fneur.2025.1631300 (PMC12833351; doi:10.3389/fneur.2025.1631300)
Supplement: Supplementary file 1 [file Data_Sheet_1.PDF]

Operated side								
**CTRL**								
	Number	FD	AD	MT	FA	AA	MA	g-Ratio
Mean Value	278,05	3,80	2,56	0,62	13,71	6,81	6,90	0,64
Median	299,00	3,49	2,20	0,60	9,54	3,81	5,28	0,67
Standard Deviation	108,34	1,75	1,46	0,22	13,50	8,24	5,69	0,11
Maximum	419,00	13,12	11,19	1,93	135,17	98,40	55,74	0,95
Minimum	70,00	0,64	0,18	0,05	0,32	0,03	0,28	0,09
**LOC**								
Mean Value	172,74	4,18	2,93	0,63	17,31	9,41	7,90	0,66
Median	173,00	3,67	2,48	0,60	10,58	4,82	5,74	0,68
Standard Deviation	28,36	2,13	1,84	0,24	18,77	12,53	6,89	0,12
Maximum	238,00	17,98	16,15	1,88	253,96	204,77	59,98	0,96
Minimum	114,00	0,92	0,18	0,05	0,66	0,03	0,31	0,07
**SYS**								
Mean Value	217,30	3,96	2,68	0,64	15,05	7,58	7,47	0,65
Median	217,00	3,58	2,39	0,64	10,05	4,47	5,74	0,66
Standard Deviation	47,94	1,87	1,57	0,23	15,13	9,54	6,11	0,11
Maximum	305,00	15,05	12,39	1,97	177,80	120,47	61,69	0,93
Minimum	136,00	0,92	0,18	0,05	0,66	0,03	0,18	0,09

Table 1: Histomorphometric parameters of the operated sciatic nerve across CTRL, LOC and SYS groups.

The table summarizes descriptive statistics (mean, median, standard deviation, minimum and maximum values) for myelinated fiber density (Number per 10^4 μm^2), fiber diameter (FD, µm), axon diameter (AD, µm), myelin thickness (MT, µm), fiber area (FA, μm^2), axonal area (AA, μm^2), myelin area (MA, μm^2), and g-ratio of the operated nerve. CTRL = control (no ASCs), LOC = local ASC administration at the repair site, SYS = systemic ASC administration via intravenous injection. All parameters represent absolute measurements from the regenerated nerve at week 14. G-ratio values across groups remained within the expected physiological range for regenerated rat sciatic nerves.

**Naïve side**							
**CTRL**							
Number	FD	AD	MT	FA	AA	MA	g-Ratio
115,20	7,52	4,90	1,31	52,42	23,75	28,68	0,63
116,00	7,80	4,85	1,38	47,76	18,50	28,27	0,64
21,53	3,19	2,49	0,46	38,84	21,60	18,54	0,09
165,00	18,28	14,99	3,25	262,50	176,50	106,98	0,84
75,00	1,01	0,37	0,18	0,80	0,11	0,53	0,24
**LOC**							
103,58	7,60	4,91	1,64	53,61	23,92	29,68	0,62
104,00	7,71	4,68	1,51	46,64	17,19	28,38	0,63
24,12	3,25	2,52	0,84	41,60	23,34	19,79	0,09
144,00	19,82	16,33	5,14	308,44	209,44	137,92	0,88
59,00	0,92	0,28	0,23	0,66	0,06	0,60	0,24
**SYS**							
94,72	8,23	5,48	1,37	61,41	28,47	32,93	0,65
92,00	8,59	5,58	1,47	57,98	24,42	33,14	0,66
12,86	3,24	2,49	0,46	41,74	23,01	19,92	0,08
125,00	18,44	15,05	2,89	267,06	177,80	119,93	0,85
78,00	1,01	0,46	0,23	0,80	0,17	0,63	0,19

Table 1: Histomorphometric parameters of the naïve sciatic nerve across CTRL, LOC and SYS groups.
The table summarizes descriptive statistics (mean, median, standard deviation, minimum and maximum values) for myelinated fiber density (Number per 10^4 µm²), fiber diameter (FD, µm), axon diameter (AD, µm), myelin thickness (MT, µm), fiber area (FA, µm²), axonal area (AA, µm²), myelin area (MA, µm²), and g-ratio of the operated nerve. CTRL = control (no ASCs), LOC = local ASC administration at the repair site, SYS = systemic ASC administration via intravenous injection. All parameters represent absolute measurements from the regenerated nerve at week 14. G-ratio values across groups remained within the expected physiological range for regenerated rat sciatic nerves.
